# The impact of emotional intelligence on workplace wellbeing among nurses and midwives: a cross-sectional study

**DOI:** 10.3389/fpubh.2026.1799655

**Published:** 2026-06-03

**Authors:** Sufia Athar, Hanen Mrabet, Charles Wesley, Kalpana Singh, Parwaneh Alshibani, Lolwa Alansari

**Affiliations:** 1 Al Wakra Hospital, Hamad Medical Corporation, Doha, Qatar; 2 Nursing & Midwifery Research Department, Hamad Medical Corporation, Doha, Qatar

**Keywords:** emotional intelligence, nurses, midwives, workplace wellbeing, job satisfaction, resilience, work-life balance, healthcare workforce

## Abstract

**Introduction:**

Despite the growing awareness of emotional intelligence (EI) as an essential component in healthcare, there remains yet a substantial lack in comprehending how EI promotes workplace wellbeing. This study examined how emotional intelligence influences job satisfaction and wellbeing among nurses and midwives in Qatar, focusing on fostering resilience in a multicultural, high-pressure setting.

**Methodology:**

This cross-sectional (convergent mixed-methods) study was carried out among nurses and midwives in a secondary hospital in Qatar after approval by the Institutional Review Board of Qatar. Our study included 121 registered nurses and midwives with at least 6 months of experience, fluent in English and Arabic, who consented and completed an online survey on seven EI domains. Descriptive statistics and inferential techniques were used for statistical analysis.

**Results:**

The overall mean EI score was 47.9 ± 7.4, with the highest proficiency in awareness of others (7.0 ± 1.7) and lower scores in emotional reasoning (5.9 ±1.8). Correlation analysis revealed a positive relationship between self-awareness and all other EI domains (*p* < 0.001), particularly emotional self-control (*r* = 0.528). While EI dimensions showed weak correlations with job satisfaction, work-life balance emerged as the primary predictor of satisfaction (*r* = 0.500, *p* < 0.001). Qualitative themes validated these results, highlighting EI as a crucial tool for stress management and team dynamics.

**Conclusion:**

This study highlights the essential role of emotional intelligence in improving workplace wellbeing, decision-making, and team cohesion among nurses and midwives, advocating for its integration as a core competency in practice and education.

## Introduction

Unlike other professions, healthcare demands not only expertise but resilience and compassion. Amidst the bustle of hectic schedules, patient overload and emerging technological innovations, resilience and compassion are gradually fading out. These implications contribute to agitation, burning out, and dissatisfaction among the healthcare staff, leaving their psychological needs unaddressed. Emotional intelligence (EI) can act as a robust tool in these scenarios, fostering recognition, reviving compassion and reinforcing resilience (1).

In multiethnic countries with diverse languages, cultural beliefs, and practices, EI is crucial for fostering effective communication, understanding, and empathy among healthcare providers and patients. EI helps bridge cultural gaps, reduces misunderstandings, and promotes respectful, person-centered care. Ultimately, it enhances trust, improves health outcomes, and supports equitable healthcare delivery across diverse populations (2).

Despite the growing evidence of its benefits, EI remains an ignored facet amongst healthcare professionals. Literature strongly supports EI, promotes emotional regulation, improving communication skills and promotes resilience and team collaboration. EI integration aids in improved patients' recovery metrics and patient satisfaction (1–3).

Gao et al., (2024), in their systematic review surveyed clinical nurses in three Chinese hospitals to assess organizational support, emotional intelligence, and work engagement. The findings demonstrated moderate levels of organizational support and emotional intelligence, with all three variables showing a positive association; emotional intelligence partially mediated the relationship between organizational support and work engagement (1). Another meta-analysis of 79 research involving 28,509 nurses found limited compassion satisfaction and rising compassion fatigue, among Asian and ICU nurses, with levels increasing between 2010 and 2019 (2). Other studies indicate that emotional intelligence can be trained, which could have a positive impact on end-of-life care (3, 4).

In recent years, studies have highlighted the significance of EI in determining occupational health across healthcare and safety-sensitive industries (5, 6). Among healthcare professionals, a higher EI has been proven to correlate strongly with better performance and resilience. Moreover, EI has been associated with better stress management, suggesting that job-related anxiety has a lesser detrimental impact on emotionally intelligent staff and makes them less prone to burnout (5, 7). Multicenter studies on nurses have indicated that those with a higher perceived EI have substantially lower likelihood of developing occupational stress, recommending integrating EI skills into nursing curriculum and continuing professional development to curb occupational stress and foster mental health (7, 8).

Research in other sectors complements these findings. It demonstrates that EI improves safety performance and accident rates in addition to mediating the relationship between occupational health and safety management practices and outcomes. This implies that workers with higher EI are better at perceiving risks, adhering to safety procedures, and maintaining concentration in hazardous environments (6).

Coalescing these results, a recent systematic review and meta-analysis of EI training programs for healthcare workers noted that all included interventions culminated in increases in EI. This supports the feasibility of EI enhancement and strengthens its role as a modifiable resource within occupational health promotion strategies, although the authors caution that methodological limitations may overestimate effect sizes (9).

Collectively, these studies converge on the verdict that EI functions as a protective factor that strengthens resilience, enhances safety in performance, and mitigates the adverse effects of occupational stress on health. Hence, this justifies the inclusion of EI assessment and training in contemporary occupational health policy and practice.

Research gaps in EI studies in healthcare include a lack of longitudinal and experimental methods to prove an association between emotional intelligence and patient or healthcare staff outcomes. There is a need for a wider range of individuals and contexts in which to investigate contextual implications on EI effectiveness. Due to the high emotional demands and safety-sensitive nature of healthcare work, addressing these gaps is central to determine whether EI can be established as a robust, evidence-based protective factor for occupational health. Initiating stronger causal links between EI and outcomes such as burnout, safety incidents, and patient satisfaction would yield clearer guidance for improving selection, training, and policy within healthcare. In this context, structured cross-sectional studies in diverse healthcare settings are necessary initiatives to map current EI levels, their association with occupational health indicators and priority groups for intervention. Therefore, further meticulous research in multifaceted healthcare settings is needed to justify systematic integration of EI assessment and training into work-related health strategies. This study investigated the relationship between EI, job satisfaction, and workplace wellbeing among nurses and midwives in Qatar, aiming to identify how emotional competencies foster resilience in a multicultural, high-pressure clinical environment.

## Methods

This cross-sectional study used a mixed-methods design and adopted STROBE standards to thoroughly investigate the impact of emotional intelligence (EI) on occupational wellbeing among nurses and midwives at a secondary hospital in Al-Wakra, Qatar. Ethical permission was acquired from Medical Research Centre, Qatar (MRC-01-24-181) and voluntary participation with informed consent ensured anonymity in online surveys (Questionnaires Attached). The study was conducted between May 2024 and April 2025. A voluntary sampling approach was used for the quantitative survey. The questionnaire was distributed digitally to all eligible nurses and midwives working at our hospital during the study period. For the qualitative component, purposive sampling was employed to select participants representing diverse roles, years of experience, and clinical areas in order to obtain a broad range of perspectives on emotional intelligence and workplace wellbeing. The quantitative component included power analysis to determine sample size, The sample size for the quantitative component was estimated using the standard single population proportion formula:

N = (*Z*^2^×*p*(1−*p*))/*d*^2^, 121 individuals completed the Genos EI brief inventory, a 14-item validated scale with strong internal consistency (Cronbach's alpha = 0.82). An online survey was implemented to collect data, which included demographic information, profession, and EI domain scores across seven core areas. To enhance the knowledge, 15 participants were purposefully selected for face-to-face semi-structured interviews that focused on substantial EI components based on the Genos model. Participants for the qualitative component were selected using purposive sampling from survey respondents who indicated willingness to participate in interviews. Semi-structured interviews were conducted by a member of the research team trained in qualitative research methods. Interviews were conducted face-to-face in a private meeting room within Al Wakra Hospital to ensure confidentiality and minimize workplace interruptions. An interview guide based on the GENOS emotional intelligence domains was used to explore participants' experiences, coping strategies, and perceptions of emotional intelligence in the workplace. Bias was minimized through diverse sampling and ensuring anonymity,

Data were analyzed using STATA version 17. Descriptive statistics were used to summarize participant characteristics. Differences between groups were assessed using *t*-tests and ANOVA where appropriate. To examine relationships between emotional intelligence domains and workplace variables, Pearson correlation analysis was performed, with statistical significance set at *p* < 0.05.

All collected data were anonymized and assigned unique identification codes. Identifiable information was removed from the dataset and stored separately on a secure institutional server at Al Wakra Hospital with restricted access to the research team only. Data were maintained according to institutional research governance policies and stored securely for future reference.

**Figure 1:**
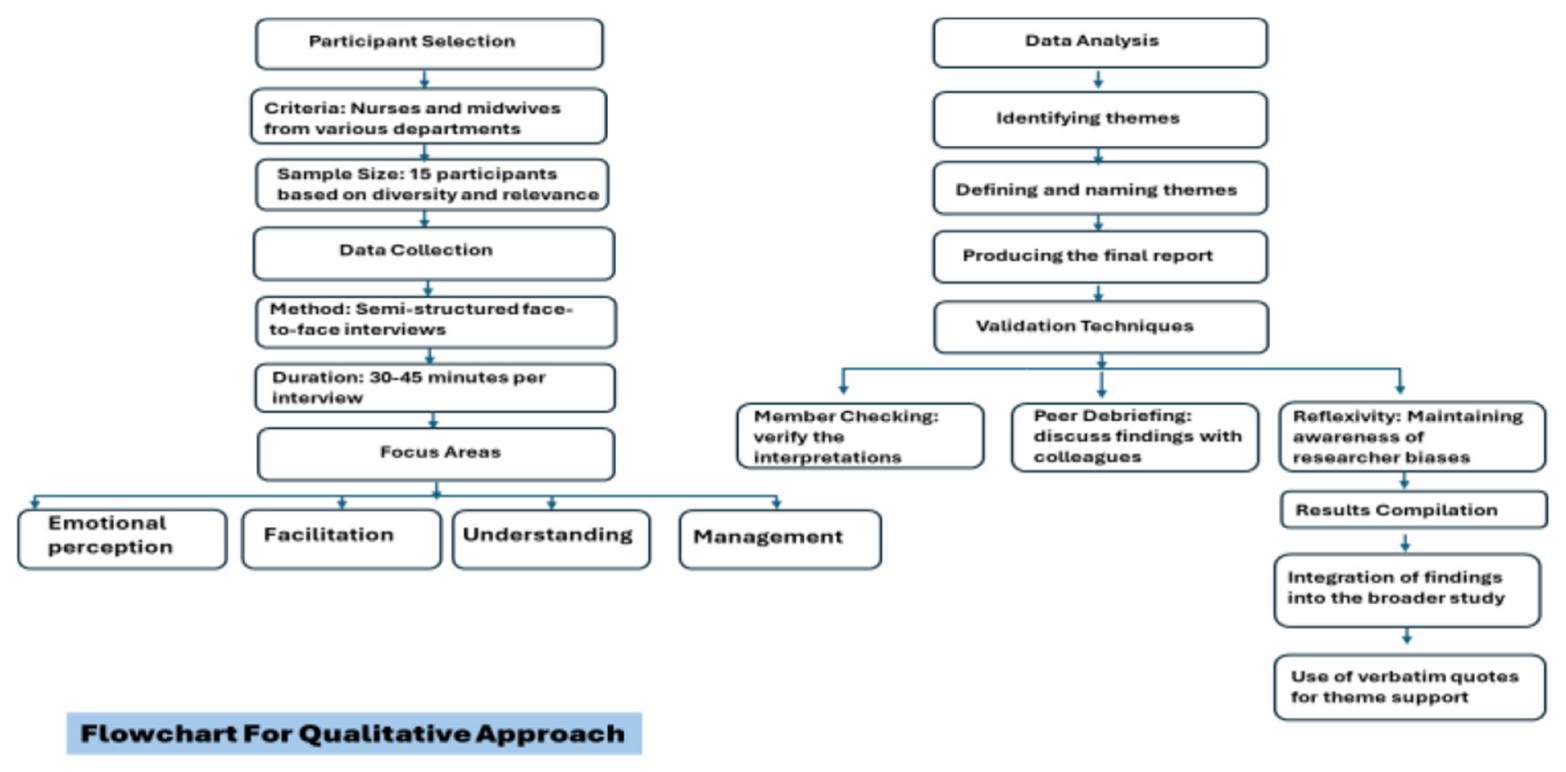
Flowchart illustrates the qualitative approach employed in the study, including participant recruitment, data collection methods), data analysis procedures and validation steps.

Thematic analysis with ATLAS.ti was used to identify and study participants lived experiences with EI, as per Braun and Clarke's framework. Member validation, peer debriefing, and maintaining an audit trail all contributed to the findings' credibility and dependability. Data coding utilized both deductive and inductive methodologies, using verbatim quotations to back up the identified themes. This technique provides a comprehensive view of how EI promotes workplace wellbeing by capturing both quantifiable associations and subjective experiences among a broad sample of nurses and midwives from various departments and experience levels (Figure 1).

## Results

## Demographics

The survey included a total of 121 participants. The age distribution among respondents was as follows: The majority of patients (86.0%) were females (as reported by participants in the survey) between the ages of 31 and 40, accounting for 63.6% of the sample population. This was followed by 24.8% of participants aged 41–50, 8.3% aged 20–30, and 3.3% aged 51–60 years. Regarding healthcare experience, 42.1% of participants had 11–15 years of experience, while 23.1% had 16–20 years of experience.

In terms of current professional roles, the majority of participants (81.8%) were registered nurses, followed by charge nurses or midwives (12.4%), head nurses or midwives (2.5%), nursing supervisors (2.5%), and urology registered nurses working within the urology clinical service. (0.8%). Regarding their educational qualifications, 81.0% of respondents had a bachelor's degree in midwifery or nursing, 10.7% had a master's degree, 7.4% had a diploma and 0.8% had a doctorate or PhD degree. 7.4% of participants received emotional intelligence training, while 92.6% did not. (Table 1).

**Table 1:** Sociodemographic characteristics of the study participants (*N* = 121).

Variables	Level	Value
**Study population (*N*)**	–	121
**Age**	20–30 years	10 (8.3%)
	31–40 years	77 (63.6%)
	41–50 years	30 (24.8%)
	51–60 years	4 (3.3%)
**Gender**	Female	104 (86.0%)
	Male	17 (14.0%)
**Years of experience in healthcare**	1–5 years	4 (3.3%)
	11–15 years	51 (42.1%)
	16–20 years	28 (23.1%)
	6–10 years	20 (16.5%)
	More than 20 years	18 (14.9%)
**Years of experience in Hamad medical corporation**	1–5 years	33 (27.3%)
	11–15 years	40 (33.6%)
	16–20 years	11 (9.2%)
	6–10 years	33 (27.7%)
	More than 20 years	2 (1.7%)
**Current professional position**	Head nurse /midwife	3 (2.5%)
	Nursing supervisor	3 (2.5%)
	Charge nurse/midwife	15 (12.4%)
	Registered nurse	99 (81.8%)
	Urology technologist	
**Educational background**	Bachelor's degree in nursing /midwifery	98 (81.0%)
	Diploma in Nursing or midwifery	9 (7.4%)
	Doctorate in Nursing or related field	1 (0.8%)
	Master's degree in nursing /midwifery	13 (10.7%)
**Have you received any training on emotional Intelligence?**	No	112 (92.6%)
	Yes	9 (7.4%)

## Survey results

Table 2 illustrates the emotional intelligence scores of 121 participants across different domains. Participants‘ average self-awareness score was 6.5 ± 1.4, indicating a moderate aptitude to recognize their own feelings. Scores ranged from three to ten. The average score for emotional awareness was 7.0 ± 1.7, suggesting participants were more conscious of others' feelings. However, there was a significant variance in scores, with some scoring as low as 1 and others as high as 10. The average score for emotional reasoning (using emotions to impact thinking) was 5.9 ± 1.8, showing slightly lower confidence in this area. Values varied from 2 to 10.

**Table 2:** Survey instrument questionnaire in accordance with the GENOS emotional Intelligence framework.

Item no.	Survey question	GENOS EI domain	Response scale
1	How often do you recognize when you are experiencing an emotion?	Emotional self-awareness	1 = Never to 5 = Always
2	How frequently do you understand why your emotions change in certain situations?	Emotional self-awareness	1 = Never to 5 = Always
3	How frequently are you able to accurately describe your feelings to others?	Emotional expression	1 = Never to 5 = Always
4	How often can you predict how you will feel in a future situation based on your current emotions?	Emotional expression	1 = Never to 5 = Always
5	How well do you comprehend the emotional expressions of others in complex scenarios?	Emotional awareness of others	1 = Never to 5 = Always
6	How often do you sense when someone needs support or assistance before they ask for it?	Emotional awareness of others	1 = Never to 5 = Always
7	How often do you notice your mood affecting how you solve problems or complete tasks?	Emotional reasoning	1 = Never to 5 = Always
8	How frequently do you identify emotions that are influencing your decision-making?	Emotional reasoning	1 = Never to 5 = Always
9	How frequently do you adjust your emotional responses to provide better support to patients and team members?	Emotional self-management	1 = Never to 5 = Always
10	How often can you differentiate your feelings of stress from feelings of frustration at work?	Emotional self-management	1 = Never to 5 = Always
11	How frequently do you successfully manage conflicts or disagreements among team members to reach a positive outcome?	Emotional management of others	1 = Never to 5 = Always
12	How often do you motivate or encourage others to remain positive under pressure?	Emotional management of others	1 = Never to 5 = Always
13	How often do you manage to remain calm and composed during medical emergencies?	Emotional self-control	1 = Never to 5 = Always
14	Can you maintain your professional demeanor under emotional stress from work demands?	Emotional self-control	1 = Never to 5 = Always
15	My work schedule allows for a balanced personal and professional life.	Work–life balance	1 = Strongly Disagree to 5 = Strongly Agree
16	I am satisfied with my role and responsibilities at work.	Job satisfaction	1 = Strongly Disagree to 5 = Strongly Agree

Table 3 depicts the range of emotional intelligence (EI) scores among nurses and midwives by age, gender, experience, role, education, and training. Nurses aged 20–30 had the highest EI scores, with a mean of 52.0 ± 6.0, while the other age groups scored between 47 and 49 points. Gender differences were minimal, with women scoring slightly higher (48.2 ± 7.5) than males (46.2 ± 7.1). Only self-awareness and awareness of others showed minor differences, which were not statistically significant (*p* < 0.05). Nurses with 1–5 years of work experience obtained the highest score (51.8 ± 5.4), whereas those with 16–20 years of experience received the lowest rating (46.6 ± 7.6) for EI. In contrast, the least experienced scored highest in emotional self-control, while the most experienced emerged as strongest in navigating the emotions of others. However, there were no significant differences between these patterns. Those with over 20 years of cumulative service got the highest score of 54.0 ± 12.7, despite the small cohort size. Notably, only awareness of others showed a significant difference across hospital service levels (*p* < 0.05), suggesting that longer tenure in the same workplace may enhance empathy.

**Table 3:** Association between sociodemographic variables and emotional intelligence domain scores.

Factor	N	Emotional self- awareness, mean (SD)	Emotional awareness of others, mean (SD)	Emotional reasoning, mean (SD)	Emotional self-control, mean (SD)	Emotional manageme nt of others, mean (SD)	Emotional expression, mean (SD)	Emotional self- managem ent, mean (SD)	Total, mean(SD)
20–30 years	10	7.3 ± 1.6	7.8 ± 1.0	6.3 ± 1.6	7.7 ± 1.3	7.9 ± 1.0	6.9 ± 1.5	8.1 ± 1.4	52.0 ± 6.0
31–40 years	77	6.5 ± 1.5	7.0 ± 1.7	6.0 ± 1.7	7.0 ± 1.6	7.5 ± 1.6	6.5 ± 1.6	7.0 ± 1.5	47.4 ± 7.4
41–50 years	30	6.2 ± 1.3	7.0 ± 1.7	5.5 ± 1.9	7.1 ± 1.5	7.9 ± 2.0	6.8 ± 1.9	7.0 ± 1.7	47.6 ± 7.6
51–60 years	4	5.8 ± 1.7	7.3 ± 2.1	6.3 ± 1.9	8.0 ± 1.2	8.5 ± 1.9	6.0 ± 1.4	7.0 ± 1.4	48.8 ± 9.5
*p*-value		0.16	0.52	0.56	0.40	0.54	0.64	0.18	0.33
Female	104	6.5 ± 1.5	7.1 ± 1.7	5.9 ± 1.8	7.2 ± 1.5	7.8 ± 1.7	6.7 ± 1.7	7.1 ± 1.6	48.2 ± 7.5
Male	17	6.5 ± 1.1	6.8 ± 1.3	5.8 ± 1.3	6.7 ± 1.5	7.1 ± 1.2	6.2 ± 1.6	7.1 ± 1.4	46.2 ± 7.1
*p*-value	–	0.84	0.45	0.75	0.21	0.14	0.34	0.96	0.31
1–5 years (healthcare)	4	7.0 ± 1.4	7.8 ± 1.7	6.5 ± 1.3	8.3 ± 1.7	8.3 ± 1.3	7.5 ± 1.0	6.5 ± 0.6	51.8 ± 4
6–10 years	20	6.3 ± 1.4	7.2 ± 1.1	5.9 ± 1.4	7.0 ± 1.3	7.4 ± 1.4	6.0 ± 1.3	7.6 ± 1.5	47.3 ± 5.9
11–15 years	51	6.7 ± 1.3	7.0 ± 1.9	5.9 ± 1.9	7.1 ± 1.6	7.6 ± 1.7	6.7 ± 1.6	7.0 ± 1.4	48.0 ± 7.5
16–20 years	28	6.4 ± 1.5	6.8 ± 1.6	5.8 ± 1.9	6.8 ± 1.5	7.4 ± 1.8	6.5 ± 1.9	6.9 ± 1.8	46.6 ± 7.6
More than 20 years	18	6.0 ± 1.6	7.3 ± 1.8	5.8 ± 1.9	7.7 ± 1.6	8.4 ± 1.7	6.9 ± 1.9	7.2 ± 1.6	49.2 ± 9.1
*p*-value	–	0.42	0.80	0.95	0.23	0.29	0.32	0.57	0.64
1–5 years (HMC)	33	6.5 ± 1.5	6.9 ± 1.5	6.1 ± 1.5	6.9 ± 1.6	7.5 ± 1.3	6.4 ± 1.5	7.2 ± 1.7	47.5 ± 7.7
6–10 years	33	6.6 ± 1.3	7.0 ± 1.5	6.0 ± 1.8	7.4 ± 1.5	7.3 ± 1.8	6.6 ± 1.4	6.9 ± 1.4	47.9 ± 7.0
11–15 years	40	6.4 ± 1.5	7.4 ± 1.6	5.6 ± 1.9	7.1 ± 1.3	7.8 ± 1.8	6.7 ± 1.8	7.1 ± 1.4	48.2 ± 6.6
16–20 years	11	5.9 ± 1.5	5.7 ± 2.5	5.7 ± 1.8	7.1 ± 2.2	8.1 ± 1.9	6.6 ± 2.3	6.9 ± 1.9	46.1 ± 10.4
More than 20 years	2	7.0 ± 1.4	8.5 ± 2.1	7.5 ± 2.1	8.0 ± 1.4	9.0 ± 1.4	6.5 ± 2.1	7.5 ± 2.1	54.0 ± 12.7
*p*-value	–	0.73	0.031	0.46	0.72	0.42	0.97	0.96	0.72

Professional roles additionally had an impact on EI scores. Head nurses/midwives and supervisors achieved slightly higher scores (51.7 ± 4.0 and 49.7 ± 4.2, respectively) than charge nurses (46.7 ± 6.6) and staff nurses (47.8 ± 7.7). Head nurses excelled in navingating the emotions of others. Bachelor's degree graduates reported the highest average EI score (48.2 ± 7.0), followed by diploma graduates (47.0 ± 9.0) and master's graduates (45.9 ± 9.2). Participants who attended EI training exhibited slightly higher overall scores (49.9 ± 11.4 vs. 47.7 ± 7.1 for the untrained). Although they scored higher on self-awareness (7.2 ± 1.8 vs. 6.4 ± 1.4), the difference was not statistically significant (*p* < 0.05) (Tables 3, 4).

**Table 4:** Emotional intelligence domain scores among study participants.

Factor	Value	Range
*N*	121	(min, max)
Self-awareness, mean (SD)	6.5 (1.4)	(3, 10)
Self-awareness others, mean (SD)	7.0 (1.7)	(1, 10)
Emotional reasoning, mean (SD)	5.9 (1.8)	(2, 10)
Emotional self-control, mean (SD)	7.1 (1.5)	(3, 10)
Emotional management of others, mean (SD)	7.7 (1.7)	(4, 10)
Emotional expression, mean (SD)	6.6 (1.7)	(2, 10)
Emotional self-management, mean (SD)	7.1 (1.5)	(3, 10)
Total, mean (SD)	47.9 (7.4)	(26, 67)

The data also evaluates the correlation matrix between factors of work-related happiness and various aspects of emotional intelligence using correlation coefficients and *p*-values. Self-awareness has a strong positive correlation with emotional intelligence, including self-awareness regulation (*r* = 0.3793, *p* < 0.001), emotional expression (*r* = 0.4803, *p* < 0.001), emotional regulation (*r* = 0.3691, *p* < 0.001), emotional utilization (r = 0.388, *p* < 0.001), and emotional management (*r* = 0.4516, *p* < 0.001). The findings indicate that higher levels of self-awareness are strongly related to other emotional intelligence skills. Additionally, there is a significant correlation with emotional self-control (*r* = 0.5281, *p* < 0.001).

Interestingly, there is a negative correlation between emotional expression and emotional self-control (*r* = −0.2248, *p* < 0.05), suggesting a potential trade-off where increased emotional expression may be associated with reduced self-control over emotions. In contrast, most correlations between emotional intelligence dimensions and work-life balance are weak and non-significant, indicating limited direct relationships in this sample.

Regarding work-related satisfaction, the most notable finding is the significant positive correlation between job satisfaction and work-life balance (*r* = 0.5007, *p* < 0.001), emphasizing that better balance between work and personal life contributes substantially to job satisfaction. Conversely, emotional intelligence dimensions exhibit weak and non-significant correlations with job satisfaction, implying that factors like emotional skills may have less influence on satisfaction compared to work-life balance. Overall, the data suggests that while emotional intelligence components are interrelated, their direct impact on work-life balance and job satisfaction varies, with work-life balance emerging as a key driver of job satisfaction in this context.

## Discussion

The study focused on the relationship of various domains of EI and its impact on the nursing staff. The results of this study demonstrate strong interrelationships among several dimensions of emotional intelligence (EI), with self-awareness as the primary variable associated with self-expression, emotional understanding, regulation, recognition, and management.

The results illustrated that EI dimensions are closely associated with self-awareness evolving as the core element. Self-awareness was closely linked to self-expression, emotional regulation, recognition and management. These findings were consistent with recent research, such as ^**^Gao et al., (2024), who observed that higher EI levels among nurses are associated with better organizational support and work engagement (4). It emphasizes the importance of self-awareness in developing emotional competencies that lead to enhanced professional performance. Cichoń et al., (2023), similarly noted that EI helps nurses develop effective coping mechanisms, which could account for the high correlations between EI facets in the current study. Individual skills contribute to emotional resilience and adaptive coping in challenging settings (2).

In contrast, EI traits have a poor or no correlation with work-life balance and job satisfaction. It can be attributed to the complexity of these notions, which can be influenced by external variables. While emotional abilities are important for dealing with stress and developing positive interpersonal relationships, they do not solely determine work-life balance or satisfaction with employment. Workload, organizational culture, managerial style, and personal circumstances all have an immense short-term impact on these outcomes. As a result, even highly emotionally intelligent people may struggle to attain a work-life balance if their circumstances are not conducive (7–10).

The negative correlation between emotional expression and emotional self-control suggests a potential dilemma or conflict between openly expressing emotions and maintaining control over them. This relationship might suggest that people who tend to express their emotions readily may find it more difficult to regulate or conceal certain feelings, particularly in high-stress situations. Alternatively, it could indicate that in certain situations, expressing emotions without appropriate self-control may be seen negatively or result in interpersonal issues, influencing perceptions of emotional regulation (11–14). More research is needed to discover whether measurement methods, cultural norms, or situational factors influence this relationship and how it varies among situations and populations.

Resilience is a term commonly associated with EI though with a different meaning. It is defined as the capacity and innate strength to tolerate stressful and challenging situations and enable individuals, teams and organizations to work even in adverse situations (15). Higher EI has been associated with higher resilience (16). It is linked to adaptive process, where EI act as a mediator between perceptiveness and adaptive processes (16).

Moreover, the emotional interconnectivity of skills indicates that strengthening EI, particularly self-awareness and regulation, can help alleviate moral distress and burnout, as suggested by Antonopoulou et al., (2024) (14). EI functions through external factors like institutional backing, leadership effectiveness, and organizational culture. It indirectly impacts work related outcomes, notably job satisfaction and engagement at workplace. Our study therefore supports that nurturing EI at workplace would enhance coping mechanisms, resilience and professionalism. It highlights that external drivers impacting EI need to be addressed by system level strategies to improve the overall impact of EI. The study accentuates the need for interventions in an organizational setting like support, supervision, balanced work and off hours and engaging culture. The results reflect that merely focusing on enhancing individual skills is insufficient without a positive work culture (14, 17).

Although EI dimensions related weakly with job satisfaction, work life balance turned out to be the predominant predictor of satisfaction. Qualitative themes sourced from interviews with nurses and midwives validated these quantitative findings with participants regularly highlighting EI as a vital tool for stress management- e.g., ”EI helps me stay calm and recover faster from rigorous shifts”, -and for enhancing team dynamics, such as, ”Understanding the emotions of my colleagues prevents conflicts and builds trust” These narratives are consistent with the existing literature implying that EI facilitates emotional regulation and social harmony within high pressure healthcare scenarios, where effective perception and management of emotions support stress coping mechanisms and reduce interpersonal conflicts (5, 18, 19).

The qualitative data further suggest that EI may influence indirectly through the enhancement of work-life balance rather than directly affecting job satisfaction, aligning with mediation models reported in literature (5, 7). This result contrasts with studies where EI directly predicts job satisfaction in less demanding settings but aligns with research in healthcare contexts where work-life balance is a key mediator in the EI satisfaction relationship due to chronic emotional demands (5). Collectively, these findings emphasize the practical significance of EI in fostering resilience and group cohesion. This indicates that targeted EI training may strengthen work-life balance and thereby indirectly improve job satisfaction among nursing staff (Table 5).

**Table 5:** Correlation matrix between emotional intelligence domains, work–life balance, and job satisfaction.

Variables	Emotional self-awareness	Emotional expression	Emotional awareness of others	Emotional self-management	Emotional self-control	Emotional reasoning	Emotional management of others	Work– life balance	Job satisfaction
**Emotional self-awareness**	1	–	–	–	–	–	–	–	–
***p*** **-value**	–	–	–	–	–	–	–	–	–
**Emotional Expression**	0.379	1	–	–	–	–	–	–	–
***p*** **-value**	< 0.001	–	–	–	–	–	–	–	–
**Emotional awareness of others**	0.480	0.056	1	–	–	–	–	–	–
***p*** **-value**	< 0.001	0.545	–	–	–	–	–	–	–
**Emotional self-management**	0.369	0.528	0.198	1	–	–	–	–	–
***p*** **-value**	< 0.001	< 0.001	0.030	–	–	–	–	–	–
**Emotional self-control**	0.102	0.531	−0.225	0.469	1	–	–	–	–
***p*** **-value**	0.267	< 0.001	0.013	< 0.001	–	–	–	–	–
**Emotional reasoning**	0.388	0.467	0.079	0.408	0.409	1	–	–	–
***p*** **-value**	< 0.001	< 0.001	0.392	< 0.001	< 0.001	–	–	–	–
**Emotional management of others**	0.452	0.568	0.181	0.512	0.457	0.446	1	–	–
***p*** **-value**	< 0.001	< 0.001	0.047	< 0.001	< 0.001	< 0.001	–	–	–
**Work–life balance**	−0.138	−0.008	−0.174	−0.042	0.084	0.056	-0.051	1	–
***p*** **-value**	0.131	0.935	0.056	0.648	0.358	0.541	0.580	–	–
**Job satisfaction**	−0.085	0.052	−0.078	0.020	0.164	0.126	0.148	0.501	1
***p*** **-value**	0.356	0.572	0.393	0.828	0.072	0.167	0.106	< 0.001	–

EI, Emotional intelligence; GENOS, genos emotional intelligence model; SD, standard deviation; HMC, Hamad medical corporation.

Despite its positive contribution, the study has a few limitations. The cross-sectional design and voluntary sampling methods of the study minimize causal inferences and introduce selection bias. While self-report measures are open to social desirability and common method variance, future broader longitudinal research with objective EI evaluation is needed to verify the findings and enhance universality. Other limitations include single institutional setting, effects of shifts on responses, personality traits, social desirability bias and organizational factors.

However, our study provides valuable insights into the impact of EI on nurses. It reflects the need for EI training for healthcare professionals. While emotional abilities are beneficial and can be improved, extrinsic drivers such as institutional support and work culture need to go hand in hand.

Ultimately, promoting a supportive environment with enhanced EI would lead to compassionate, effective nursing care. Emphasizing both personal growth and organizational commitment is key to transforming healthcare experiences for both nurses and patients. Only through this combined approach can we truly elevate the standards of nursing practice and patient outcomes. Though EI seems related to compassionate nursing care, further longitudinal and experimental studies are needed to validate the results.

## Data Availability

The original contributions presented in the study are included in the article/supplementary material, further inquiries can be directed to the corresponding author.
